# The Crush the Crave Quit Smoking App and Young Adult Smokers: Qualitative Case Study of Affordances

**DOI:** 10.2196/mhealth.9489

**Published:** 2018-06-08

**Authors:** Laura L Struik, Joan L Bottorff, Neill Bruce Baskerville, John L Oliffe

**Affiliations:** ^1^ Propel Centre for Population Health Impact Faculty of Applied Health Sciences University of Waterloo Waterloo, ON Canada; ^2^ Institute for Healthy Living and Chronic Disease Prevention University of British Columbia Kelowna, BC Canada; ^3^ School of Health and Exercise Science Faculty of Health and Social Development University of British Columbia Kelowna, BC Canada; ^4^ School of Public Health and Health Systems University of Waterloo Waterloo, ON Canada; ^5^ School of Nursing Faculty of Applied Science University of British Columbia Vancouver, BC Canada

**Keywords:** smartphone, smoking cessation, young adult, qualitative research, social theory

## Abstract

**Background:**

Mobile phone apps have emerged as a promising way to reach young adult smokers, given their high mobile phone ownership rates and openness to receiving cessation support via digital technologies. Although emerging evidence indicates that quit smoking apps are an effective way to reduce smoking among young adults, lacking is formative evaluative research that captures the perspectives of end-users.

**Objective:**

The objective of this study was to contribute insights toward understanding how young adults interact with the Crush the Crave (CTC) app, and how this interaction shapes young adults’ smoking cessation experiences and practices, with consideration of the influence of gender.

**Methods:**

Semistructured interviews were conducted with 31 young adult CTC end-users. Guided by sociomateriality theory and an affordances approach, data were inductively analyzed to derive thematic findings in relation to the impacts of CTC on quit efforts, and to expose the underlying affordances (mechanisms) that lend to these outcomes. Findings were grouped according to the 4 design components of CTC: credibility, social support, task support, and dialogue support.

**Results:**

The credibility component of CTC played an important role in harnessing the trust of young adults because it afforded them promise in relation to its potential effectiveness in assisting them with quitting smoking. The social support component lent to various end-user practices and experiences that rendered this aspect as the weakest component in supporting quit efforts. Although most functions situated in the task and dialogue support components were found to be helpful, there were a few affordances in CTC that resulted in negative experiences, notably weaning from smoking. Gender-related influences were also evident. For example, young men preferred to control and self-manage their quitting and, therefore, did not engage with functions that afforded journaling or reminding to stay on track. Women, in contrast, were more likely to benefit from these affordances.

**Conclusions:**

An affordances approach is productive for gaining an in-depth understanding of how mobile apps interact with end-users to lend to particular outcomes. The study findings have implications for developing and improving apps for helping young adults quit smoking, as well as apps that target other health behaviors. Productive affordances may also serve as a framework for leveraging apps for smoking cessation.

## Introduction

### Background

Finding effective strategies to assist young adults with quitting smoking is a priority in light of their high smoking rates [1,2] and low uptake of available cessation resources [3]. Given young adults’ ubiquitous presence in the mobile phone market [3], many researchers have turned their attention to mobile phone apps as a promising way to reach young adult smokers [4]. There are now over 500 smoking cessation apps available between the Apple iOS and Google Play App stores, which have been met with enthusiasm by health care consumers according to the large number of downloads [5]. Young adults particularly indicate a desire for cessation support via apps compared with other interventions [6-8].

**Figure 1:**
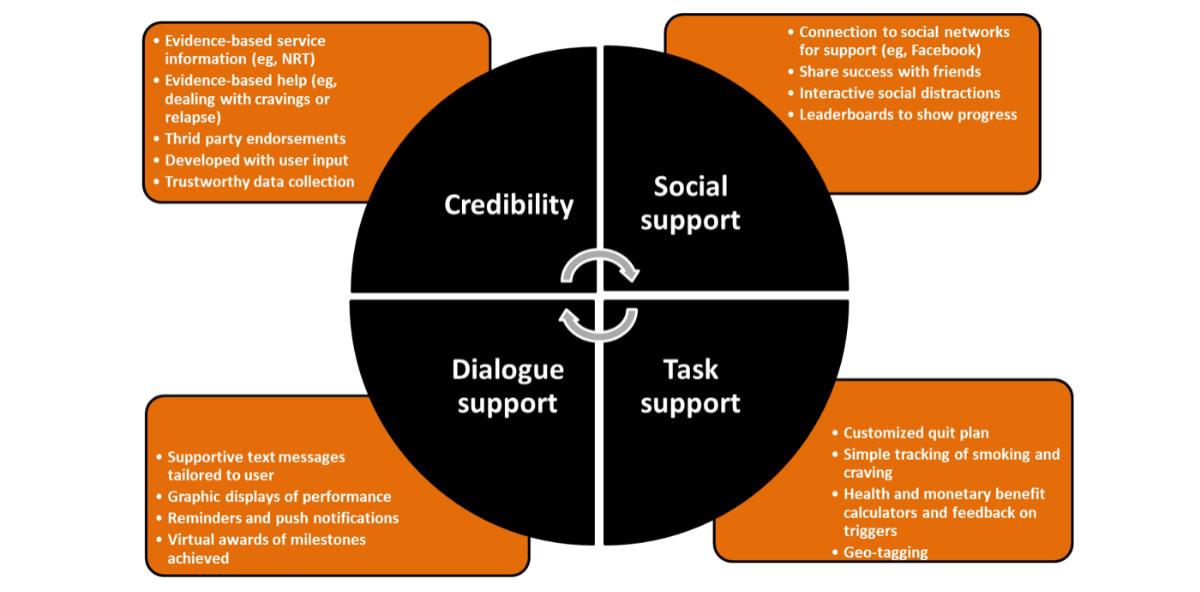
Crush the Crave design components. NRT: nicotine replacement therapy.

In the Crush the Crave (CTC) randomized controlled trial (RCT) [9], the CTC mobile phone app, which aims to help young adults quit smoking, was evaluated. CTC was developed in 2012 by a multi-sectoral team as an evidence-informed quit smoking app for young adults aged between 19 and 29 years. Underpinned by the US Clinical Practice Guidelines for quitting smoking [10] and principles of persuasive technology for behavior change [11], CTC includes 4 key design components: credibility (eg, backing by credible agencies), social support (eg, a Facebook community), task support (eg, tracking of cravings and smoking habit), and dialogue support (eg, virtual awards that credit performance toward reaching goals; Figure 1). The RCT compared CTC with a control condition (smoking cessation print materials) to assess efficacy of the app for helping young adults quit smoking. This study focuses on a qualitative exploration of the experiences of trial participants who interacted with the CTC. Trials evaluating smoking cessation apps are emerging, with recent systematic reviews summarizing preliminary evidence that smoking cessation apps are effective [12,13]. Although this quantitative evidence is important, qualitative data that capture end-users’ perspectives are lacking. Given that these interventions are situated in the daily lives of smokers, understanding end-users’ perspectives and the mechanisms by which these apps may promote behavior change is critical for adjusting and leveraging future apps focused on quitting smoking [14,15]. In addition, despite established evidence that gender-sensitive approaches are needed for smoking cessation interventions, and that electronic health (eHealth) cessation interventions that include gender-sensitive approaches positively influence receptivity to and use of the interventions [16-18], the empirical base regarding the influence of gender-related factors or ways to incorporate a gender-sensitive approach into mobile-based smoking cessation interventions is under developed.

### Objectives

The purpose of this study was to contribute insights toward understanding how young adults interact with the CTC app, and how this interaction shapes young adults’ smoking cessation experiences and practices, with consideration of the influence of gender.

## Methods

### Design

This study complemented the RCT that was underway to evaluate the CTC app where data were collected at baseline, 3 months, and 6 months [9]. Young adults who had been assigned to the intervention group were recruited in this study. Ethics approval for this study was obtained from the University of British Columbia (Okanagan campus) Behavioral Research Ethics Board (certificate #H15-00466).

A qualitative case study design [19] was chosen, with CTC as the case. In addition to being paradigmatically flexible [20], this approach enables flexible boundaries around what entails the case, is ideal for investigating phenomena on which little is known, and promotes a commitment to intense, contextually minded, and the holistic study of a case. Given that the app functions and is influenced by young adults’ contextually laden day-to-day lives, the case study was an ideal method for investigating the interactions between CTC and young adult end-users. Congruent with this methodology, data collection was qualitative in nature, and analysis followed the framework approach, which aims to draw descriptive and explanatory conclusions clustered around themes [21].

### Participants

Using a purposive sampling strategy, young adults aged between 19 and 29 years, who used CTC and were either recent quitters or still smoking, were sought. Recruitment included young adults participating in the RCT who completed the 6-month questionnaire and selected *yes* to receiving information about this qualitative companion study. These participants provided their contact information (email and phone number) and, therefore, recruitment was conducted via email, phone calls, and text messaging. Altogether, 31 young adults provided informed consent and participated in this study. Participants were between 20 and 29 years (mean 24, SD 2.72), a little less than half were female at 42% (13/31), they were predominantly white at 71% (22/31), and from the province of Ontario at 48% (15/31). Most completed some postsecondary or college education at 67% (21/31), followed by high school at 13% (4/31), less than high school at 10% (3/31), and a University degree at 10% (3/31). Most participants (24/31, 77%) were still smoking at 6 months after using the CTC app.

Regarding end-user experiences with CTC (see Table 1), when using the app, the majority of participants used the app 1 to 3 times per month. However, only 23% (7/31) were still using CTC at the time of interview. In addition, 45% (14/31) of the sample reported being satisfied with the app. All features of the app were used, except 2 (online resources and my map). The majority of participants reported using the cigarette and craving tracking features the most. The crave community features (Facebook and Twitter) were the least used and least liked.

### Data Collection

Data were collected using semistructured individual telephone interviews with young adults. The interviews were guided by sociomateriality theory in that interview questions aimed to uncover the actionable items that CTC enabled or constrained to reach the goal of quitting smoking. In this regard, interview questions were focused on *how* CTC enabled or constrained their quit smoking goals (eg, *Please tell me about a time or times that you used [function used] and how it helped you in your quit efforts.*).

All interviews were audiotaped and lasted between 30 and 80 minutes. Study participants received an honorarium (Can $50 per interview) to acknowledge the time spent on the study.

### Theoretical Approach

To guide this study, Leonardi’s [22] sociomateriality theory was employed. This theory was developed in response to observations that qualitative research on the use of digital technologies tends to focus on end-user perceptions, although the technological aspects of these innovations remained unexamined [23,24]. Sociomateriality offers a way to overcome established opposition between social and material determinism, considering both technological tools (material agency) and individuals (social agency) as 2 components of the same underlying phenomenon [25].

Operationalized through the concept of affordances, Leonardi’s sociomateriality theory foregrounds actionable items (affordances) that result from the interactions (also called imbrication—joining together) between humans and technological tools. Although an app can do many different things (material agency), what it does do is only provoked through an end-user (human agency), and this may vary according to the context of an end-user. Therefore, an affordance is an underlying mechanism as a result of a sociomaterial relationship (eg, an app’s functions and a young adult), which subsequently lends to particular experiences and actions. In this regard, productive and unproductive affordances of a mobile-based health intervention for a population may be revealed through a sociomateriality lens. Using sociomateriality, affordances of CTC are highlighted in what follows to explain Canadian young adults’ experiences and practices in relation to quitting smoking with CTC.

### Data Analysis

Data collection and analysis were conducted simultaneously, so that emerging themes could be further investigated in subsequent interviews. Sampling continued until data were repeated and representative coverage of emergent themes were saturated [26]. Findings repeated by 6 or more interviews served as the starting point of saturation [27]. To guide data analysis, the framework approach by Ritchie and Lewis [21] was employed, which is hallmarked by a series of interconnected stages (familiarization, identifying an analytic framework, indexing, charting, and mapping and interpretation) that enable the researcher to move back and forth between the data until a coherent account of the phenomenon is developed [28].

The interviews were first transcribed verbatim and uploaded in the qualitative data analysis software program NVivo version 10 (QSR International Pty Ltd, Burlington, MA, USA). Data from interviews with young women were kept as a separate dataset from those with young men to enable the lead author to compare and contrast young women’s and men’s experiences and identify notable gender-related influences in the datasets and findings. After a detailed reading of the transcripts in their entirety to become familiar with the data, a preliminary coding framework was developed using data from the first 4 interviews with young women and young men. In addition to coding young adults’ descriptions of how and why they used the app, the coding framework was informed by sociomateriality theory, in which codes and categories were inductively derived for young adults’ practices and experiences in using CTC, and then the affordances that lent to them. The frameworks for each dataset were then reviewed and approved by all authors. Indexing was accomplished by coding major themes and subsequent subthemes in relation to the analytical framework in NVivo, which was revised or added to as new data were collected.

**Table 1:** Crush the Crave (CTC) end-user experiences.

CTC usage item		Response, n (%)
**Frequency of use**		
	Never	2 (6)
	1-3 times per month	23 (74)
	Once a week	2 (6)
	2-3 times per week	2 (6)
	Daily	2 (6)
**Overall satisfaction**		
	Not at all satisfied	9 (30)
	Not very satisfied	7 (23)
	Somewhat satisfied	7 (23)
	Satisfied	5 (16)
	Very satisfied	3 (10)
**Would use CTC again**		
	Yes, still using it	7 (23)
	Yes, but not using it now	11 (35)
	No	13 (42)
**Features used**		
	Cigarette tracker	14 (45)
	Craving tracker	11 (35)
	Distractions page	3 (10)
	Awards page	6 (19)
	My progress page	12 (39)
	Health calculators page	9 (29)
	My map feature	0 (0)
	Leaderboard	1 (3)
	My quit plan page	5 (16)
	Information pages	3 (10)
	Online resources	0 (0)
	Quitline	1 (3)
	Crave community (Facebook, Twitter)	1 (3)
	None of the above	6 (19)
**Most helpful features**		
	Cigarette tracker	10 (32)
	Craving tracker	6 (19)
	Distractions page	2 (6)
	Awards page	2 (6)
	My progress page	5 (16)
	Health calculators page	4 (13)
	My map feature	0 (0)
	Leaderboard	0 (0)

Charting was accomplished by developing summaries of the interview data in tables and figures. The final stage of analysis, mapping and interpretation of the data, involved comparing the interviewee’s responses within each assigned category and subcategory. At this time, finalized themes and subthemes were established. This final analytical framework was then transferred into a table. Representative quotes were selected to illustrate key themes and subthemes.

## Results

### Young Adult’s Use of Crush the Crave

Young adults described being motivated to use the app because it was a novel quit support, with CTC being the first cessation app that most of them had interacted with. Young adults often expressed exhaustion with the lack of success or negative side effects associated with using other quit strategies and welcomed CTC as a new alternative. In addition, participants described alternative quit aids, namely, nicotine replacement therapies, Champix, and acupuncture as unfulfilling because they targeted one aspect of smoking, primarily nicotine addiction, and they described needing an intervention that addressed the habitual nature of smoking in their daily lives. CTC was, therefore, described as a much-needed, relevant quit support.

In relation to their use of the app, participants said that they primarily used the app to reflect on their smoking behavior at the end of the day or week when they had “down time.” Despite the app being designed to intercept cravings and smoking in real time, young adults described their day-to-day life as not always amenable to having their mobile phone constantly with them. Therefore, even if they had cravings or smoked cigarettes throughout the day, they were not always able to document and reflect on these events until they had some free time.

Young adults also described waning engagement with CTC, where they used it most intensely when it was first downloaded, but their usage diminished over time. Young adults described 3 reasons for this: first, it aligned with their quit smoking trajectory—the less they smoked, the less they engaged with the app, particularly those who opted to quit cold turkey; second, they became disinterested in the app because of technological glitches and usability issues (eg, freezing, only functions with an Internet connection, and resources often located outside the app), offsetting the convenience of having the app at their side; and third, they abandoned their quit attempts.

### Affordances of Crush the Crave

In this section, affordances that resulted from young adults’ interactions with each component of CTC (credibility, social support, task support, and dialogue support) and young adult’s subsequent experiences and practices in relation to quitting smoking are presented. Table 2 presents representative quotes to support the findings. Differences reflected in young women’s and men’s conversations are highlighted throughout.

### Credibility

The credibility design component of CTC related to the app being developed and supported by credible agencies and research institutions. Knowing that the app was supported by these agencies afforded young adults promise—CTC was a quality quit smoking resource worthy of young adults’ trust. As a result of this affordance, participants described trusting the app’s intent to help them quit smoking, as well as its potential to influence a positive smoking cessation outcome.

### Social Support

The social support design component refers to the parts of the app that aimed to provide young adults with opportunities to harness support from new and existing social networks. Interviews revealed that the social support aspects in the app were least helpful with smoking cessation. Young adults’ experiences were the result of the following affordances: *constrained identity protection; competition with others;* and *constrained coparticipation,* which shed light on their low usage rates of the crave community features.

#### Constrained Identity Protection

The features and functions that aimed to provide social support, particularly the social media components of CTC, were described by participants as discordant with how they wanted to harness social support for quitting smoking. Although they did want support with cessation, they explained that posting on the “open” group Facebook page would reveal their smoking status and efforts to quit to others, especially to those in their personal networks. Young adults often spoke about changing social norms that increasingly reflected an intolerance of smoking to justify decisions to conceal their smoking and protect themselves from others’ judgment. Furthermore, they said that if they posted about quitting, pressures to succeed would mount, and if they were not successful, then they feared being further judged as failing or weak.

In keeping with their efforts to protect themselves, young adults primarily practiced “lurking” and avoided posting on the social media channels made available through CTC. The few young adults who were open to posting about their quit smoking efforts on Facebook said that they would only post about successes in their quit smoking efforts when they were confident about their smoke-free status.

#### Inhibited Competition

The leaderboard function was discussed as a function that inhibited productive competition with other CTC end-users, in that the competition embodied defeating each other rather than getting to know and support each other. As a result, participants found the competition discouraging, as well as divisive, which was magnified by the anonymous nature of the leaderboard, inhibiting being able to connect with each other.

#### Constrained Coparticipation in Quitting

The CTC function to encourage end-users to find a “quit buddy” was confusing to most participants. They did not know if it should be a fellow smoker who was also trying to quit. As most young adults did not have access to someone who was also ready to quit, they often bypassed this function in the app. Young adults who did try to use the support of a quit buddy said that it was not helpful because the quit buddy was often not quitting the same way that they were.

**Table 2:** Representative quotes.

Design component and affordance		Young adults’ experiences and practices	
**Credibility**			
	Promise		Trust in app intent: *It made it seem more legit. Like it was actually something making you try to quit smoking instead of, you know, maybe the other ones have ads in them and they try to make money off them. But this one clearly isn’t. It’s a little more genuine, you know? (male, nonsmoker, P24)*
**Social support**			
	Constrained identity protection		Feelings of vulnerability: *And if you don’t [quit] like people might get on you and nag you or be disappointed. I don’t need that. I’ll be disappointed in myself, that’s enough. (male, smoker, P21)*
			Selective posting: *You know, I’m gonna kinda keep it to myself and work away at it. And then, once I have quit for good, then maybe I can go and say like, this is where I’m at this point in the app. I’ve quit smoking completely, or it’s been 100 days or whatever the case may be if I wanna share my milestone or something like that. But [its]…nothing I would use on a regular basis for sure. (female, smoker, P26)*
	Inhibited competition with others		Discouraging and divisive: *Someone whose successful and quit smoking isn’t any better than someone that’s struggling with it. Like, no, I didn’t-I don’t like that aspect… it just makes someone feel bad. (male, nonsmoker, P10)*
	Constrained co-participation		Unhelpful: *I tried to do the quit buddy thing but the people that I was having as quit buddies were not as serious about quitting as I was so. (male, nonsmoker, P17)*
**Task support**			
	Visibility of the benefits of quitting		Perception shift – health implications became relevant: *Yeah, I would check that one a lot because it would keep coming up and like showing I’m this close to being…back to like a nonsmoker for this aspect of my health, or this aspect...I guess it was more of a motivation to quit because some of them it takes like years to get back to a non-smoker health state and that just really made me think about what I was doing. (female, smoker, P14)*
	Documentation of smoking behavior		Raised awareness of smoking habit: *I thought I knew how much I was smoking but this [smoke button] gave me a reality check….it was neat to see how much I was actually smoking. (male, smoker, P23)*
	Mapping out smoking behavior		Irrelevant information: *For me, it’s definitely not geographic triggers that make me wanna smoke. It’s more like…like day-to-day triggers of either stress, or commuting traffic, and like bad news. You get bad news broken on you or you had a bad day it’s like, yeah I’d be more tempted to pick up that cigarette. Those type of things. More than the, oh like I’m located at 24 Sussex here and, I have a temptation. (male, smoker, P3)*
	Journaling about smoking behavior		Unhelpful (men): *No [I wouldn’t put in my triggers]. I would just put the craving in but the triggers, my main triggers were coffee, which I have quite a few a day, friends, drinking, after a meal.. It’s the first thing I wanna do. (male, smoker, P4)*
			Proactive about triggers (women): *I loved…how you could go in and track [journaling] what your triggers were so I could actually start to notice my triggers and stay away from them. I’m craving when I’m with friends drinking, or you know, my son’s acting up, and stuff like that. So I could see what was actually causing me to want to smoke and I could actually try and change them. [For example], it was my friend’s birthday party and I knew we were all going to be in the same house and she smokes in her house. So I had to come prepared. I brought gum, I brought mints, I brought everything I could think of because that was one of my main times when I smoke a lot…when I’m drinking. (female, smoker, P25)*
	Weening from smoking		Procrastination: *I actually think that that’s [quitting abruptly instead of weening off smoking] the way that it has to be done….Like it’s almost like exercising and diet. You can’t say you’re gonna start next week. It’s really like right now or never…. Like that’s one thing that I think was the flaw in CTC. (female, smoker, P28)*
	Counseling through cravings		“Backpocket” support: *I didn’t personally call [the quit line] but it was just—I think it was more of just knowing that like, if at any time, if I didn’t have someone to talk to or if there wasn’t a feature on the app that I could use, [the quitline] was kind of like in my back pocket right? Like if I absolutely 100% needed to make a call, I could. (male, nonsmoker, P17)*
**Dialogue support**			
	App reminding		Irritating: *I completely ignored them [notifications]. Actually, I’m pretty sure I had the notifications that were from the app all turned off. It just felt like a pop up, like another thing for me to click close on throughout the day. I completely paid no attention to it. (male, nonsmoker, P10)*
			Motivation to quit (men): *I found it [notification reminders?] was almost like having my girlfriend there, in a good way. So you’re like, oh I haven’t done this in two days, I didn’t even realize, but my phone just reminded me. Better keep it going. (male, smoker, P3)*
	Recognition of ability		Confidence boost: *It [what??] was just a reminder just to say like how good you’re doing. And if there’s nobody physically around you to be like, oh you’re doing such a great job, then [the app] did it for you. Like yeah, keep up the good work, you’ve been this many days without a craving or whatever else. It was kinda like a motivational boost. (female, smoker, P11)*
	Visibility of quit efforts		Motivation to quit: *It [what?] was like a visual of my day of smoking. And every day, you’d look at it, it went down and down and down, like it got better every day. So it was like a motivational thing to just look, like positive reinforcement. (female, smoker, P11)*
			Discouragement: *If you're having a bad day or a couple of bad days, seeing it on [the app] as a reflection [of your bad days] just like kicks you in the face even more, you know? (female, smoker, P22)*

### Task Support

The task support functions were essentially aimed at supporting young adults in accomplishing their “task” of quitting smoking (eg, through tracking their behavior and identifying their triggers). Overall, young adults were enthusiastic about the helpfulness of the task support functions. Various experiences and new ways of dealing with their smoking behavior were achieved through the following affordances: *visibility of the benefits of quitting, documentation of smoking behavior, journaling quit smoking experiences, counseling through cravings, entertainment, and weening from habit.*

#### Visibility of the Benefits of Quitting

Participants described how the calculating functions in the app afforded them context to the benefits of quitting smoking. They described a shift in their perceptions about the impact of smoking on their health when, eg, they could actually see with one of the app calculators the amount of cigarette tar in lungs decreasing as fewer cigarettes were smoked. They explained that seeing improvements that were otherwise invisible (eg, tar in lungs) made them realize that smoking is a relevant concern for them, rather than something they do not have to worry about until later on in life.

#### Documentation of Smoking Behavior

Documentation of smoking behavior was afforded via the smoke and crave buttons. Although using this feature enhanced awareness of smoking patterns, some young adults, particularly young men, were not as inclined to document their smoking behavior as women, often describing it as “cumbersome” to have to manually enter their data.

#### Mapping Smoking Behavior

Although the map function was intended to geographically map out where smoking or cravings occurred, young adults did not see this feature of the app as helpful or relevant. They explained that geography in and of itself was not a trigger and that their smoking triggers were situational.

#### Journaling Quit Smoking Experiences

Journaling, whereby young adults were prompted to reflect on their behavior when they smoked in response to triggers, was afforded through the feedback function. In relation to this aspect of the app, young men were adamant that the triggers feedback function lacked benefit because they smoked when they chose to smoke, not because they were triggered. As they already knew what prompted them to smoke, recording their triggers was perceived as unhelpful. Young women, however, described how journaling lent to a more proactive approach toward dealing with cravings. They learned how to anticipate when and where they would have a craving and would implement measures to prevent smoking in these situations.

#### Counseling Through Cravings

Access to quitline counseling through the app was constructed by end-users as a “last resort.” Although most indicated they would not likely use counseling, this feature was described as “nice to have” in the app if they “really” needed it. Despite comfort in knowing quitline counseling was available, actual use of the quitline was associated with discomfort. Many stated that they did not want to get on the mobile phone with someone they did not know. The few who did consult with the quitline, spoke despairingly about their experiences. Although it did help them overcome their craving, one young women (smoker) stated that talking to a quitline counselor made her “feel like an addict,” and a young man (nonsmoker) complained about being “warned about how bad smoking is for you” from the quitline counselor.

#### Entertainment

The craving distractions in the app afforded young adults’ entertainment during moments of boredom that prevented them from smoking. Although there were several types of distractions in the app (games, videos, music, and social media), young adults preferred games because they “kept their hands busy.” On the other hand, young adults found YouTube videos and music less effective, because their hands were available to hold a cigarette and smoke while they watched a video or listened to music.

#### Weening From Habit

Although most young adults chose to quit cold turkey (particularly young men), there were a few who chose to use the quit plan laid out in the app. However, the weaning afforded via the quit plan was primarily described as something that promoted procrastination in relation to becoming smokefree. Young adults who chose to quit cold turkey stated that they avoided the quit plan for the same reason.

### Dialogue Support

The dialogue support design component related to aspects of the app that aimed to positively reinforce the decision to quit smoking. Interviews with young adults revealed that, overall, the dialogue support features lent to positive experiences by young adults, as they engaged in quitting smoking with a few exceptions. These experiences were the result of the following affordances: *app reminding*, *personalized reminding, recognition of ability*, and *visibility of progress.*

#### App Reminding

Reminders about quit smoking progress and the benefits of quitting, afforded via the push notifications, were described by most end-users as a source of irritation because every app now has push notifications. To avoid having to “swipe away” notifications to keep their mobile phone “clean,” some end-users simply turned off the notifications feature. Reminding via the push notifications was well received by a few men but not women. Several men asserted that the notifications helped to keep them motivated in relation to their quit smoking goals.

#### Personalized Reminding

Reminders of personal motivations to quit were afforded via the personalization functions in the app (eg, providing end-users with the option to upload personal photos and quotes to motivate them in their cessation efforts). Young women found this method of reminding as motivational. Young men, however, described a general disinterest in customizing apps and technological platforms in general.

#### Recognition of Ability

Many participants reported that the awards offered through the app afforded them recognition of their quit smoking efforts, which was described as a “confidence boost.” The receipt of an award affirmed that they were able to reach cessation milestones and essentially enhanced their confidence in accomplishing their ultimate goal of quitting smoking. Women, however, sometimes found the awards discouraging when they were struggling to reach them.

#### Visibility of Quit Efforts

Young adults described the visibility of their quit smoking efforts via the progress page in the app, whereby a graph displays cravings and cigarettes smoked month to month, week to week, day to day, and hour by hour, as motivational. Seeing a decrease in their cravings and smoking was an incentive to keep going. A few young women and men, particularly women, however, experienced discouragement and guilt on seeing this page when they were not making steady progress.

## Discussion

### Principal Findings and Implications

In this qualitative study, we examined the interaction between young adults and CTC for quitting smoking through an affordances lens, a relatively novel approach to mobile health (mHealth) research. This approach enabled a detailed understanding of the underlying affordances that influenced young adults’ quit smoking efforts while using CTC. Both productive and unproductive affordances for aiding in young adults’ quit smoking efforts were identified. Although all affordances in relation to the social support component of the app were unproductive, with a few exceptions, the other components of the app were largely productive. These findings serve as a strong foundation for identifying practical steps that can be taken to modify and strengthen CTC and smoking cessation apps more broadly.

The gaps in knowledge in relation to the underlying mechanisms of mobile-based health interventions, particularly in the area of tobacco control, are what motivated the use of sociomateriality theory and an affordances approach. Although the use of sociomateriality theory and an affordances approach is a relatively novel approach to mHealth research, its use in examining CTC enabled a detailed understanding of the underlying mechanisms (affordances) of the sociomaterial relationship (CTC app and young adult) that influenced young adults’ quit smoking efforts. Researchers have described this knowledge as a “black box” because most eHealth evaluations are focused on outcomes rather than the underlying factors and mechanisms [29-31]. As demonstrated in the findings of this study, the specificity and depth that such an approach provides are of utmost value for understanding how to move these interventions forward. This is because the underlying mechanisms of these interventions that lend to both positive and negative experiences and practices in relation to health behavior change are brought forward—articulating practical and tangible ways in which these interventions may be modified to strengthen effectiveness and be scaled up. In relation to CTC, productive affordances can be capitalized upon to enhance uptake and impact (eg, visibility of quit smoking benefits, recognition of end-user efforts, and reminding of end-users’ progress), and unproductive affordances of these interventions can be addressed (eg, developing CTC for gradual quitting). In short, the use of sociomateriality theory and an affordances approach removes a lot of guesswork in relation to linking up end-users’ experiences and practices to improvements in an eHealth intervention.

By focusing on affordances (action possibilities that result from imbrication between an app and a population) rather than on the app’s features and functions, mechanisms that result from the interaction between the app and an individual are highlighted. In this regard, the success of an app does not lie in the features in and of themselves but in the potential action possibilities that the features and functions embody for health behavior change. An affordances approach reveals which action possibilities of an intervention have the most positive effect in relation to ongoing end-user engagement, end-user appeal, and the target behavior change. Once productive affordances are identified, then features and functions can be designed to capitalize on these affordances. Indeed, the productive affordances revealed in this study may serve as a framework for improvement of existing smoking cessation apps and development of future apps.

### Productive Affordances

Productive affordances identified in this study provide useful directions for capitalizing and scaling up some aspects of CTC, as well as other apps for smoking cessation. A few noteworthy affordances should be considered. For example, backing by credible agencies resulted in affording young adults promise that the app would indeed help them quit smoking. Despite hundreds of quit smoking apps being available, CTC had an edge among the young adult population because of its backing. It is, therefore, recommended that those who are developing evidence-informed apps make the institutions that support the app explicit in their marketing.

Also noteworthy is the affordance of documentation of smoking behavior (eg, cigarettes smoked or cravings experienced), which enabled young adults to be more self-aware of their smoking and craving patterns. Young adults’ desire to track their behavior was reflected in their high use of the app’s tracking features. The popularity of tracking features in smoking cessation apps is also supported in the literature and has been identified as a key reason by end-users to stay engaged with smoking cessation apps [32,33]. The importance of affording young adults with the ability to document their smoking behavior is brought forward in light of recent suggestions that successful behavior change is dependent on engagement with an intervention [34,35]. Considering promising developments for affording “smart” documentation via sensors and wearables, this productive affordance may be leveraged to strengthen engagement, while at the same time reducing end-user data input burden.

Affording young adults’ visibility of quit benefits through the health calculators, which provided tailored information about the health benefits of reducing and stopping smoking, countered some optimism bias in relation to the predicted effects that smoking had on them. This optimism bias among young adult smokers is recognized in the literature [31-33]. For example, in an evaluation of the potential use of health-related factors to motivate smoking cessation among college students, it was found that almost half of smokers thought that their health was better or the same as their nonsmoking counterparts, almost all of the smokers did not think that their health was affected by smoking, and nearly half thought that quitting would bring little to no benefit to their health [36]. More recently, young adult smokers were found to demonstrate optimistic bias in relation to their risk perception and health-related behaviors for cancer, respiratory disorders, and cardiovascular diseases [37]. In a qualitative study about how young adults initiate smoking, young adults could not recall the health risks of smoking, struggled to assess the likelihood of developing health problems from smoking, and rarely saw health risks as personally relevant, often citing the tobacco industry’s argument that the role of smoking in disease could not be easily delineated [38]. The findings of this study reveal how affording visibility in relation to the benefits of quitting via physiological markers (eg, lung improvement) is a breakthrough accomplishment of mobile-based interventions in young adult smoking cessation, and smoking cessation in general, and may be leveraged as embodied motivation for cessation.

Although affording end-users’ visibility of their quit efforts and recognition via the gamification features of the app (eg, awards and progress statistics) was effective in motivating young adults to continue with their smoke-free efforts, some young adults found these functions discouraging when they struggled or relapsed. The literature indicates that although points, statistics, and badges are an important element of gamification features for motivating behavior change, motivation is only 1 of the 3 important elements of gamification for health behavior change [39]. In addition to motivation, capability and behavioral triggers must also be considered and integrated into gamification features (eg, through problem solving, storytelling, and fantasy) [40]. Given that quitting smoking is a process known to frequently encompass struggles and relapse [41], the importance of enhancing self-efficacy for cessation and awareness of smoking triggers should be understood as key. Unless an individual is on a straightforward success trajectory, which is seldom the case, it is possible to see how a focus on gamification to strengthen motivation is unreliable as a sole influence for smoking cessation. This is consistent with recent research indicating that an exclusive focus on points, rewards, leaderboards, or badges has an unreliable impact on behavior change because it only strengthens motivation [39]. The findings of this study extend knowledge in this area by highlighting how the affordances of visibility of quit efforts and recognition for gamification features are important but insufficient.

### Unproductive Affordances

Unproductive affordances identified provide useful directions for improving the CTC app. Although the social support component of the app was designed to provide young adults with opportunities to harness social support, in keeping with previous findings [37], wherein the CTC Facebook posts were primarily posted by a CTC moderator rather than a young adult smoker, young adults seldomly posted on the CTC Facebook page. The findings of this study indicate that this component afforded constrained identity protection, inhibited productive competition, and constrained coparticipation. Preservation of and efforts to promote a positive self-presentation pervaded young adults’ discussions in relation to the social support design component of the app, especially in relation to the CTC Facebook page. Young adults’ avoidance of posting on the CTC Facebook page aligns with literature, describing health communication on social media as being at odds with the need to present oneself as a positive, appealing community member [42,43]. According to researchers who examined self-presentation strategies employed by young adults on Facebook [43], this platform is widely used to enhance one’s self-presentation versus derogate oneself (eg, presenting struggles or negative events). Interestingly, however, Bereket-Bojmel and colleagues [43] found that those who did engage in self-derogation were rewarded with social network support (demonstrated through increased numbers of likes and comments). In a study that examined outcomes of positive versus honest presentation on Facebook, it was found that honest self-presentation had an indirect effect on well-being through perceived social support [44]. Although a private (vs the public CTC Facebook page) Facebook group or forum may address some concerns and promote more honest self-presentations, more research is needed to understand how to effectively provide opportunities for social support in the context of young adult smoking cessation via eHealth.

The influence of normative pressures also appeared to be a factor in young adults’ avoidance of the social support functions in CTC. Shifting social norms, whereby smoking has become increasingly stigmatized, underpinned reluctance to harness support for cessation efforts within their own networks as well as on the CTC Facebook page. Unintended consequences of tobacco control initiatives include stigmatization of smokers [45,46]. It is well recognized in the literature that smoking stigma leads to social isolation, decreased self-esteem, shame, perceived negative judgment, and increased stress [47]. The findings of this study suggest that the effects of smoking stigma extend into mobile environments, creating a barrier to the use of social media as a social support tool in apps for cessation. Engaging end-users in future app development is needed to ensure social support features are acceptable to end-users, reflecting their needs and preferences for identity protection, rather than risking reinforcement of smoking stigmas.

The quit buddy function did not afford coparticipation in quitting smoking and was, therefore, also unsuccessful. Young adults cited issues with finding someone who also wanted to quit at the same time, already having an established support network, and discomfort in harnessing support through a quit buddy. Similar difficulties were reported with a text messaging–based intervention for young adults that included a quit buddy component (quit buddy was another intervention participant) [8]. End-users reported problems with their buddy's availability, including being in different stages of the quitting process, on different schedules or in different time zones, already having established support, or being uncomfortable with the idea of a quit buddy [8]. Although evidence suggests that a buddy system works well in the context of a smokers’ clinic [48], this study’s findings add to emerging evidence that a buddy system has yet to effectively translate to mobile cessation interventions, and specifically brings attention to coparticipation as the key mechanism for this type of social support to effectively work.

Affording weening from smoking through a gradual quit plan was also unproductive, with young adults primarily opting to quit abruptly once they downloaded the app without any gradual reductions in smoking. This finding complements Ubhi and colleagues’ [49] insights, with 50% of their sample consisting of young adults, who found that most (84%) participants opted to quit on the date of their registration versus gradual quitting using the Smokefree28 app. The young adults in this study found that gradual quitting made them procrastinate about actually quitting and may explain emerging evidence that quitting abruptly is better suited to those ready to quit, whereas gradually quitting aids those who are not ready to quit [50]. Future developers of cessation apps for young adults should include an option for quitting abruptly and work with end-users to identify tools and resources to support this approach to quitting.

### Gender-Related Influences

There were important differences in young women’s and men’s preferences in relation to affordances. Young women expressed an appreciation for affordances that helped them become more self-aware and develop new coping skills (eg, journaling) and personal reminders about why they wanted to quit smoking. In contrast, most young men explained that they did not need reminding about why they should stop smoking and did not feel the need to journal about their smoking. Men’s preference for app features that reinforced autonomy and ability to quit on their own appeared to reflect masculine norms and ideals (eg, men are strong and independent) [51], and therefore provide promising directions for developing apps that appeal to young men who smoke.

The CTC health calculators afforded women and men meaningful and impactful information about the health effects of smoking. This finding is particularly important in relation to men who often ignore health-related information, rendering them a hard-to-reach population to engage in health behavior change [52]. The “visibility” into personal health afforded via the data presented by health calculators, however, seemed to capture men’s attention and interest in improving their health by offering objective measures by which men could track their progress. Perhaps, these personal health data play into men’s preferences for autonomy and self-monitoring and management to support behavior change. Mobilizing masculinities for positive health behavior change has recently become a focus in men’s health research [53,54]. Although more research is needed, affording visibility of personal health data presents as a promising way to mobilize positive health practices in men via mobile phone apps.

### Limitations

There are limitations in using self-report to capture young adults’ interactions with the CTC app in their everyday lives. There may be affordances and constraints that were not captured during the interviews. Some young adults were interviewed up to a year after they entered the RCT study, potentially limiting their ability to recall their experiences. To minimize this limitation, reflective questions were posed during interviews to assist participants in recalling events and experiences. Although the sample included individuals who successfully quit smoking as well as those who did not, the latter were the largest group. Further research is needed to determine if affordances identified in this study are directly linked to the app’s efficacy in supporting cessation. In addition, the sample was primarily white, limiting the applicability of the findings to other population groups. Given the similarities between this study sample and the RCT sample [9], however, this study findings appear to hold strong transferability to the young adult smokers’ sample included in the RCT study and, therefore, Canadian young adult smokers in general.

Finally, although the speed of technology and changes in the sophistication of end-users are challenges in claiming the application of eHealth research to future interventions, the focus on affordances lent through the app versus the actual features of the app provides a strong grounding from which to improve existing and future innovations. For example, affording visibility of quit smoking benefits is likely to remain an important goal even as eHealth and end-user sophistication progresses. Although new affordances will emerge with new innovations and new contexts, the productive and unproductive affordances found in this study will remain ever-important considerations in future mobile-based smoking cessation interventions targeting young adults.

### Conclusions

This is one of the first studies in the area of eHealth research to employ sociomateriality theory. As such, this study makes a significant contribution to addressing the “black box” of knowledge in relation to how and why aspects of eHealth interventions succeed or fail. Through this study, both productive and unproductive affordances of CTC for young women’s and men’s quit smoking efforts were revealed. The productive affordances identified in this study can serve as a beginning framework for improving CTC and developing scalable health behavior change apps.

## Abbreviations

CTC: Crush the Crave
eHealth: electronic health
mHealth: mobile health
RCT: randomized controlled trial
